# The Structural Role of Elastic Fibers in the Cornea Investigated Using a Mouse Model for Marfan Syndrome

**DOI:** 10.1167/iovs.16-21358

**Published:** 2017-11

**Authors:** Tomas L. White, Philip Lewis, Sally Hayes, James Fergusson, James Bell, Luis Farinha, Nick S. White, Lygia V. Pereira, Keith M. Meek

**Affiliations:** 1 Structural Biophysics Research Group, School of Optometry and Vision Sciences, Cardiff University, Maindy Road, Cardiff, United Kingdom; 2 Vision Science Bioimaging Labs, School of Optometry and Vision Sciences, Cardiff University, Cardiff, United Kingdom; 3 Department of Genetics and Evolutionary Biology, University of São Paulo, Rua do Matão, São Paulo, Brazil

**Keywords:** cornea, elastic fiber, fibrillin, Marfan syndrome, microfibrils

## Abstract

**Purpose:**

The presence of fibrillin-rich elastic fibers in the cornea has been overlooked in recent years. The aim of the current study was to elucidate their functional role using a mouse model for Marfan syndrome, defective in fibrillin-1, the major structural component of the microfibril bundles that constitute most of the elastic fibers.

**Methods:**

Mouse corneas were obtained from animals with a heterozygous fibrillin-1 mutation (*Fbn1*^+/−^) and compared to wild type controls. Corneal thickness and radius of curvature were calculated using optical coherence tomography microscopy. Elastic microfibril bundles were quantified and visualized in three-dimensions using serial block face scanning electron microscopy. Transmission electron microscopy was used to analyze stromal ultrastructure and proteoglycan distribution. Center-to-center average interfibrillar spacing was determined using x-ray scattering.

**Results:**

*Fbn1*^+/−^ corneas were significantly thinner than wild types and displayed a higher radius of curvature. In the *Fbn1*^+/−^ corneas, elastic microfibril bundles were significantly reduced in density and disorganized compared to wild-type controls, in addition to containing a higher average center-to-center collagen interfibrillar spacing in the center of the cornea. No other differences were detected in stromal ultrastructure or proteoglycan distribution between the two groups. Proteoglycan side chains appeared to colocalize with the microfibril bundles.

**Conclusions:**

Elastic fibers have an important, multifunctional role in the cornea as highlighted by the differences observed between *Fbn1*^+/−^ and wild type animals. We contend that the presence of normal quantities of structurally organized elastic fibers are required to maintain the correct geometry of the cornea, which is disrupted in Marfan syndrome.

The optical and biomechanical properties of the human cornea are largely governed by the collagen-rich stroma, a layer that represents approximately 90% of the total thickness. Within the stroma, the specific arrangement of superimposed lamellae confers requisite mechanical properties,^1^ while the spatial arrangement of individual collagen fibrils within the lamellae enables transparency.^2,3^ As well as collagen, elastic tissue has a fundamental biomechanical role in many dynamic tissues,^4^ for example, lungs and the intima of blood vessels, where it routinely experiences strains in excess of 100% in response to changes in blood pressure and vasoactivity.^5^ Similarly, the sclera contains an elastic fiber system^6,7^ allowing the eye to deform slightly as a result of internal and external pressure, before regaining its original shape. Despite the cornea being part of the outer tunic of the eye, along with the sclera, the presence of elastic tissue in the corneal stroma has been overlooked in recent years. The development of more advanced imaging techniques, such as serial block-face scanning electron microscopy (SBF SEM), has enabled this area to be revisited. We recently have used this technique to characterize a complex network of elastic fibers in human cornea.^8^ These fibers consist of bundles of microfibrils, the main component of which is the fibrillin-1 protein.^9,10^ In the human cornea they appear as elastin-containing sheets at the limbus before extending into the peripheral and central stroma as narrower fibrillin-rich microfibril bundles, most of which appear to be devoid of elastin cores. These microfibril bundles run parallel to the surface of the cornea and are concentrated in the posterior stroma, immediately anterior to Descemet's membrane.^8^ Serial block-face scanning electron microscopy also has been used to demonstrate the presence of fibrillin-rich microfibril bundles in the mouse corneal stroma,^9^ although their functional role remains unclear.

Marfan syndrome (MFS) is an autosomal dominant disease of connective tissue caused by mutations in the *FBN1* gene encoding the fibrillin-1 protein.^11,12^ The condition results in ocular, skeletal, and cardiovascular abnormalities, caused by altered biomechanical properties in tissues containing elastic material. The most common symptom seen in the eye is ectopia lentis, induced by biomechanically weak ciliary zonules that hold the lens in dynamic suspension. Mouse models for MFS have been developed with variable phenotypic severity to study the pathogenic mechanisms underlying the disease.^13–15^ Lima et al.^15^ developed a mouse model (mgΔ^loxPneo^) with a mutant *Fbn1* allele that produces an internally truncated fibrillin-1. Heterozygotes present defective microfibrillar deposition, which results in kyphosis and cardiovascular abnormalities (deterioration of the aortic wall and aneurysms) equivalent to those of MFS patients.

The corneas of mgΔ^loxPneo^ mice have not been characterized previously. In an attempt to elucidate the functional role of elastic fibers in the cornea, we have used a combination of SBF SEM, transmission electron microscopy (TEM), optical coherence tomography (OCT) microscopy and x-ray scattering to examine corneas from the mgΔ^loxPneo^ mouse model for MFS in comparison with wild type (WT) controls. Corneas of mgΔ^loxPneo^ mice are referred to as *Fbn1*^+/−^ throughout this study. It also should be noted that the term “elastic fibers” here refers to bundles of fibrillin-rich microfibrils, or oxytalan fibers, as opposed to true elastic or elaunin fibers, which contain fibrillar and amorphous components.

## Methods

### Mouse Eyes

Whole eye globes from *Fbn1*^+/−^ and WT mice were obtained from the Department of Genetics and Evolutionary Biology, University of São Paulo, Brazil. Development of the *Fbn1*^+/−^ mouse model has been described previously.^15^ *Fbn1*^+/−^ mice, along with WT controls, were euthanized at 3 months of age. Whole eye globes were removed immediately following death and fixed in 0.5% paraformaldehyde before being transported to the United Kingdom in a box cooled with ice packs and stored at 4°C. All animal procedures were performed in accordance with the ARVO Statement for the Use of Animals in Ophthalmic and Vision Research.

### OCT Microscopy

**Figure 1:**
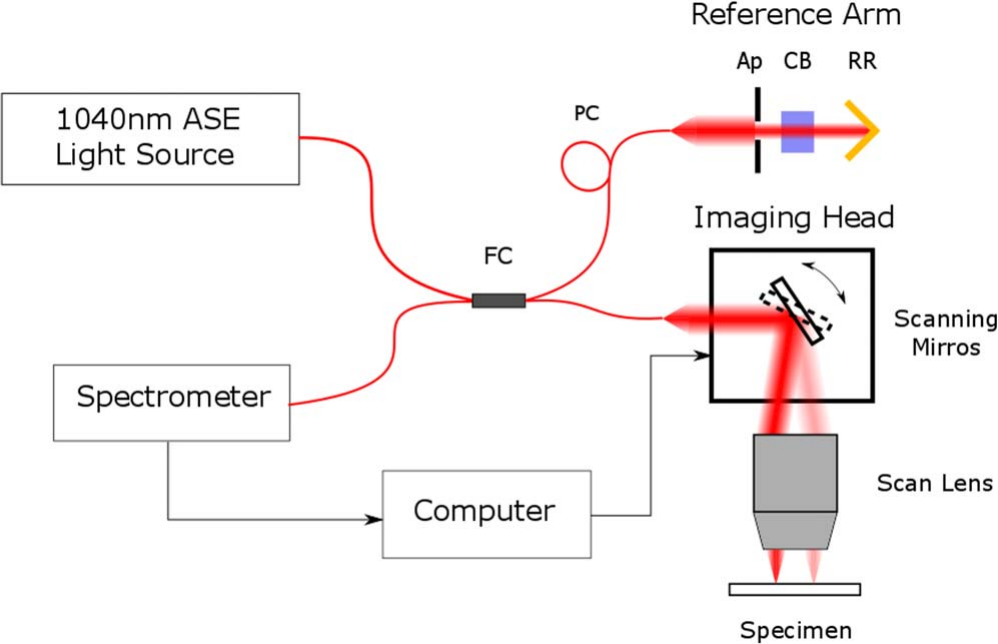
Optical coherence tomography microscopy setup. Illumination from an amplified spontaneous emission light source with a central wavelength of 1040 nm and a bandwidth of 70 nm (1-M-ASE-HPE-S; NP Photonics, Tucson, AZ, USA) was coupled through a 2 × 2 optical fiber coupler (FC; FOBC-2-64+/−100-20-L-H64f-2; AFW Technologies, Hallam, Victoria, Australia) to an imaging head and reference arm. The imaging head comprised an achromatic off-axis parabolic reflector (not shown) to collimate the fiber output beam to approximately 2 mm diameter (RC02APC-P01; Thorlabs, Ely, UK), close-coupled 2D (XY) optical scanners (6210HBM60/6102103R; Cambridge Technology Division, GSI Group GmbH, Muenchner, Germany) and a broad-band NIR telecentric scan lens (LSM02BB; Thorlabs). The reference arm consisted of a polarization controller (PC; FPC560, Thorlabs), a second reflecting collimator (not shown; RC08APC-01; Thorlabs), an adjustable aperture (Ap), a precision NIR retroreflector (RR; 1 Arcsec Gold, Edmund Optics, York, UK) and a glass compensation block (CB; LSM02DC; Thorlabs) to correct for NIR dispersion in the scan lens. Reflected light from the specimen and reference arm was combined in a spectrometer.^58^ The camera has been upgraded to a 47khz Goodrich SU-LDH-1.7 (UTC Aerospace Systems, Arlington, VA, USA).

Eyes from eight WT and nine *Fbn1*^+/−^ mice were imaged using a near infrared (NIR) bespoke OCT microscope (Fig. 1). The digitized spectra of reflected light from every point of a 2D (XY) scan over the specimen were processed into a 3D volume image by spectral resampling and Fourier transformation. The instrument was controlled by LabView (National Instruments, Newbury, UK) software and spectra and image data were processed using MATLAB (Mathworks, Cambridge, UK) and ImageJ software.^16^

A small well made from mounting putty was created on a glass slide to hold the eye in the correct position for scanning (with the cornea facing upwards). The eye was hydrated with drops of 0.5% paraformaldehyde before being imaged. Data sets of 1000 images at 1000 × 1000 pixel resolution (axial scaling of 2.66 μm, lateral scaling 3.09 μm per pixel in air) were obtained from eye globes, focused on the cornea and anterior chamber. Data sets were reconstructed into 3D models using Amira 6.0.1 software (FEI, Mérignac, France).

Central corneal thickness was calculated by firstly taking the average intensity of approximately 10 images from the center of each data set. The images had an axial scaling of 2.66 μm per pixel in air; therefore, for any part of the image that is in the tissue, the scaling is reduced by the refractive index of the material (1.4 in the case of the mouse cornea).^17^ The number of pixels from the anterior to the posterior surface at the center of the cornea was measured and converted to actual thickness using the following formula:

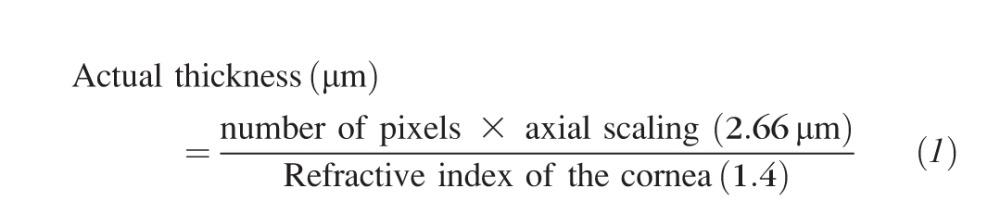



**Figure 2:**
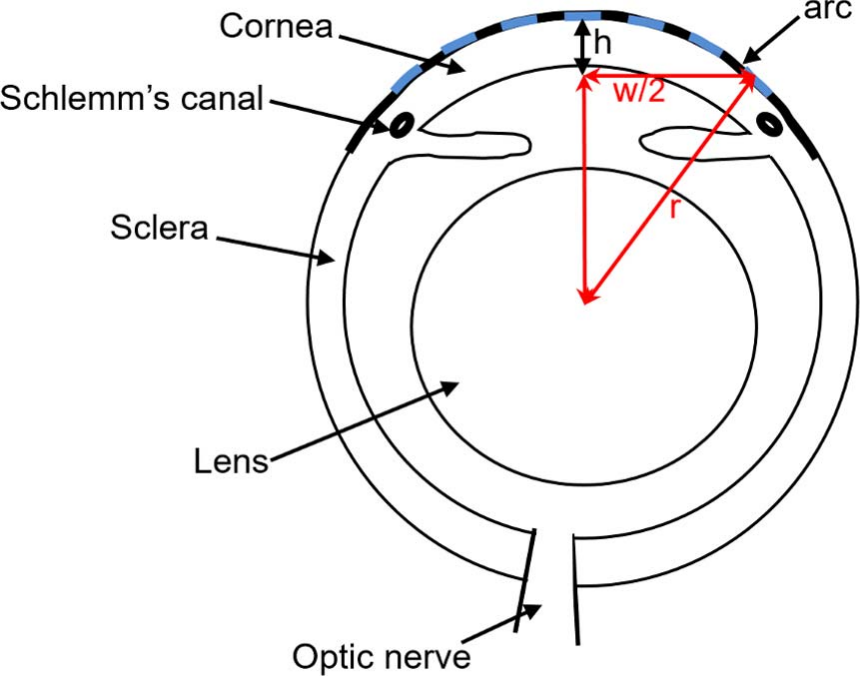
Schematic of a mouse eye to illustrate the parameters used in the calculation of corneal radius of curvature from optical coherence tomography images. The radius of curvature was calculated by applying Pythagoras theorem to the red triangle. The height (h) of the arc was maintained at 300 μm in all cases, with only the width (w) varying between specimens.

The radius of curvature was measured from the arc formed at the anterior surface of the central cornea (Fig. 2) using the following equation:

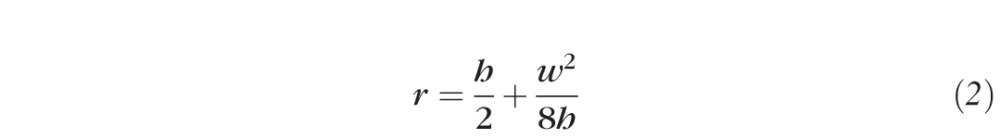



The height (*h*) of the arc being measured for each sample was consistent at 300 μm to ensure that the same size arcs were being measured, with only the width (*w*) varying. All thickness and radius of curvature measurements were performed using ImageJ software.^16^

### Serial Block Face Scanning Electron Microscopy

Three mouse corneas from each group were dissected from whole globes, cut into small segments, and fixed in modified Karnovsky's fixative^18^ (2.5% glutaraldehyde and 2% paraformaldehyde in 0.1M cacodylate buffer at pH 7.2) for 2 hours. Tissue was processed according to methods described previously.^8^ Briefly, this consisted of postfixation with 1% osmium tetroxide, incubation in 0.5% tannic acid, staining with 2% aqueous uranyl acetate, dehydration in an ethanol series, and further staining with 2% ethanoic uranyl acetate and a saturated solution of lead acetate^19^ before being embedded in CY212 (TAAB) epoxy resin. Specimens were examined using a Zeiss Sigma VP FEG SEM (Carl Zeiss Meditec, Jena, Germany) equipped with a Gatan 3View2 system, where data sets of up to 1000 images were acquired every 50 nm at 4000 × 4000 pixel resolution. Three-dimensional (3D) reconstructions of data sets were created with Amira 6.0.1 software, using automated and semiautomated thresholding.

Elastic fiber quantification throughout the entire central stroma was carried out on one cornea from each group to estimate the amount of elastic tissue in a normal mouse cornea, and to discover the extent of elastic fiber reduction in the *Fbn1*^+/−^ cornea. Additionally, obtaining these data provided 3D information throughout the entire depth of the cornea. Quantification of elastic fiber density was done as detailed previously for human corneas.^8^ Data sets were acquired through the entire depth of WT and *Fbn1*^+/−^ corneas at 2000 × 2000 pixel resolution. Each full-thickness set of data was analyzed in groups of approximately 250 images (equating to a depth of approximately 12.5 μm), where percentages of tissue volume occupied by elastic fibers in each group were calculated using Amira 6.0.1 software.

### Transmission Electron Microscopy

Embedded tissue that had been stained en bloc for SBF SEM also was used for TEM. A Leica UC6 ultra-microtome (Leica Biosystems, Wetzlar, Germany) was used to cut 90 nm-thick gold sections that were floated on distilled water before being mounted on copper grids and visualized using a JEOL 1010 TEM (JEOL, Welwyn Garden City, UK).

### Cupromeronic Blue Stain

Small segments of tissue from two WT and two *Fbn1*^+/−^ corneas fixed in Karnovsky's fixative were washed in 25 mM sodium acetate buffer containing 0.1 M MgCl_2_ (pH 5.7) for 3 hours at room temperature on a rotator. Segments were stained with the 25 mM sodium acetate buffer containing 0.1 M MgCl_2_ and 0.05% cupromeronic blue at room temperature overnight. Tissue was rinsed (3 × 5 minutes) with the sodium acetate buffer (without cupromeronic blue) before being exposed to 0.5% aqueous sodium tungstate (3 × 10 minutes) and 50% EtOH + 0.5% sodium tungstate for 15 minutes.^20,21^ This was followed by dehydration through an ascending ethanol series (70%, 90%, 100% EtOH) and propylene oxide. The tissue was incubated in a 1:1 mixture of propylene oxide and epoxy resin (araldite monomer CY212, DDSA hardener, BDMA accelerator) for 1 hour before 100% epoxy resin infiltration for 1.5 days. Cornea segments were placed into plastic molds with fresh epoxy resin and polymerized at 60°C for 36 hours. Ultrathin (approximately 90 nm) gold sections were cut, collected on copper grids, and allowed to dry overnight. Sections were counterstained with 1% aqueous uranyl acetate for 15 minutes, washed with distilled water for 5 minutes, and visualized with TEM.

### X-Ray Scattering

Four WT and four *Fbn1*^+/−^ corneas, obtained from individual mice, were dissected from whole globes that had been fixed and stored in 4% paraformaldehyde. Small-angle x-ray scatter patterns were collected on beamline I22 (Diamond Light Source, Oxfordshire, UK), using an x-ray beam measuring approximately 200 × 200 μm with a wavelength of 0.1 nm. Before x-ray exposure, each cornea was wrapped in catering cling film to prevent dehydration and placed inside an airtight Perspex sample holder containing two transparent Mylar sheet windows. The sample holder then was positioned on the beamline such that the center of the anterior surface of the cornea was perpendicular to the incident x-ray beam. X-ray scatter patterns resulting from a 0.4-second exposure were collected at 0.2 or 0.3 mm intervals along the vertical transect of each specimen. X-ray scatter patterns were collected on an x-ray detector positioned 6 m behind the specimen. Our previous small-angle x-ray scattering studies have shown that the hydration of mouse corneas examined in this way does not significantly change.^22,23^

The x-ray scatter patterns were analyzed as described previously^24,25^ using a custom-made graphic user interface written in MATLAB (MathWorks). Each pattern was radially integrated to generate an x-ray intensity profile, from which background scatter was removed by fitting and subtracting a power-law background function. Scattering patterns were centered using the pattern of powdered silver behanate, and calibrated against the 67 nm meridional spacing of hydrated rat tail tendon. Average interfibrillar Bragg spacing (IFS) of corneal collagen fibrils was determined from the calibrated position of the first-order equatorial reflection. Measurements of IFS were made at each sampling site along the vertical axis of the cornea.

### Statistical Analysis

Tests for normality and equal variance were performed using Shapiro-Wilk and Levene's tests, before an independent *t*-test being done to determine whether any differences existed between *Fbn1*^+/−^ and WT corneas for central corneal thickness, radius of curvature, elastic fiber density, and IFS. Correlation between the corneal thickness and radius of curvature variables was tested using Pearson's correlation test. *P* < 0.05 was determined as statistically significant. All statistical testing was done using IBM SPSS software (IBM, Armonk, NY, USA).

## Results

### Corneal Thickness and Radius of Curvature Measurements

**Figure 3:**
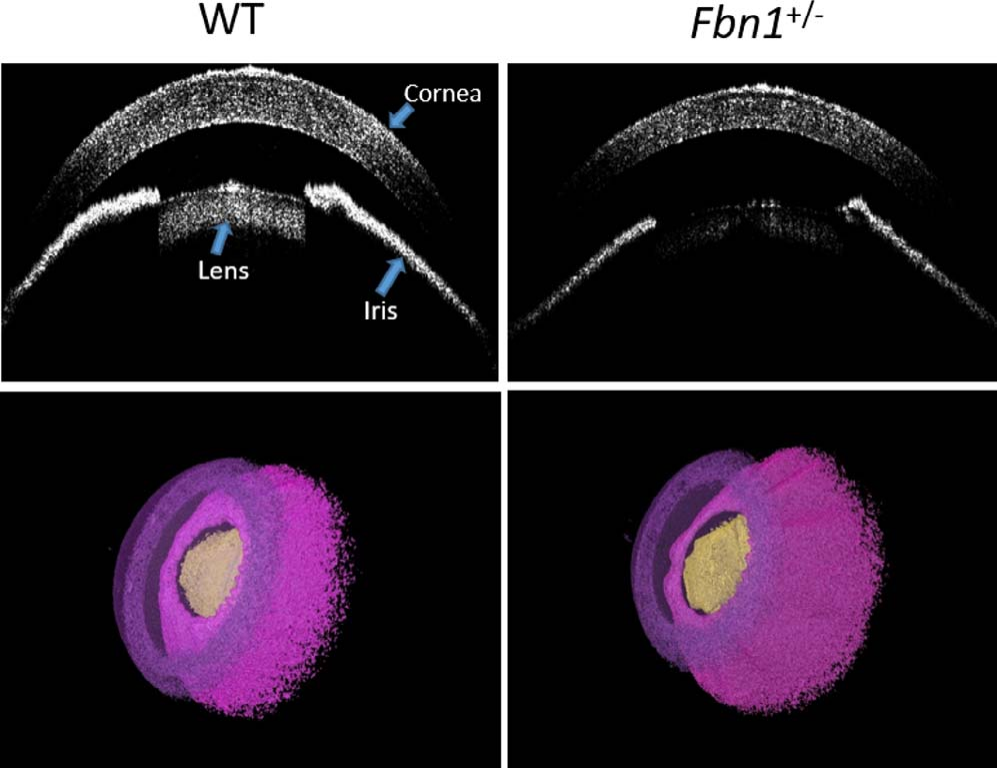
A series of 1000 optical coherence tomography images were obtained from each eye and reconstructed into 3D models. By viewing individual images (top), it appeared as though the WT cornea (left) was thicker and more curved than the Fbn1^+/−^ cornea (right). The cornea (purple), lens (yellow), and iris (pink) were represented in 3D (bottom) for both groups.

Individual images taken from the center of each OCT microscopy data set revealed a difference in central corneal thickness between WT and *Fbn1*^+/−^ corneas (Fig. 3). Additionally, *Fbn1*^+/−^ cornea appeared to be flatter compared to the more curved WT. The center-mid corneas were segmented, along with the iris and pupil. As a result of poor contrast, the peripheral cornea was omitted from the 3D reconstructions.

**Figure 4:**
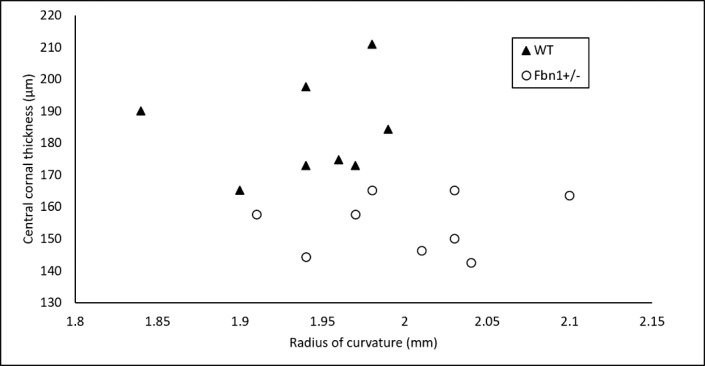
Central corneal thickness plotted against radius of curvature. Each point on the graph represents measurements from a single mouse cornea (8 WT, 9 Fbn1^+/−^). Average central corneal thickness was significantly lower (P < 0.001) in Fbn1^+/−^ corneas (154.8 μm) compared to WT controls (183.6 μm). Conversely, average corneal radius of curvature was significantly higher (P < 0.05) in Fbn1^+/−^ animals (2.00 mm) compared to WT equivalents (1.94 mm).

Using the OCT microscopy images, central corneal thickness and radius of curvature were measured for WT and *Fbn1*^+/−^ corneas (Fig. 4). Independent *t*-tests showed that WT corneas were significantly thicker than *Fbn1*^+/−^ corneas (*P* < 0.001), with an average thickness of 183.6 μm (±15.2), compared to 154.8 μm (±9.1) in the fibrillin-1–deficient mice. Wild type corneal thickness reached >190 μm, whereas *Fbn1*^+/−^ corneas often were <150 μm, meaning a difference of approximately 40 μm (approximately 1/3 of the stromal thickness in mice). Radius of curvature also was significantly different between the two groups (*P* < 0.05), with an average radius of 1.94 mm (±0.049) in WT, and 2.00 mm (±0.057) in *Fbn1*^+/−^ corneas. This was more subtle than the difference in thickness, with many radii lying between 1.94 and 1.99 mm. Statistical analysis revealed that no significant correlation existed between radius of curvature and corneal thickness (*P* = 0.20).

### 3D Arrangement and Quantification of Elastic Fibers

**Figure 5:**
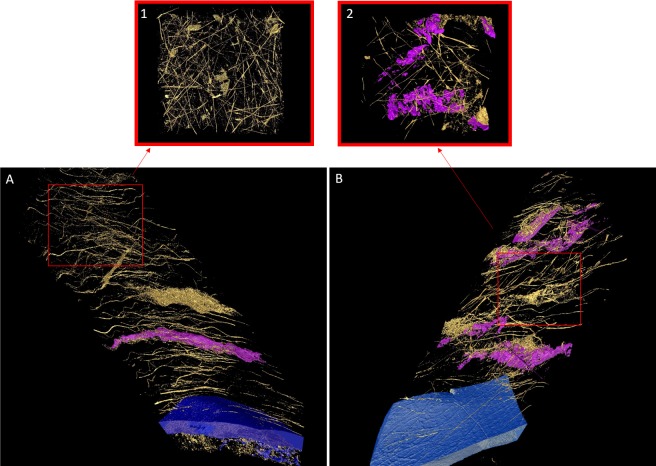
The 3D arrangement of elastic fibers (gold) in the first approximately 50 μm of stroma above Descemet's membrane (blue) in WT (A) and Fbn1^+/−^ (B) mouse cornea. Additionally, some of the residing keratocytes have been segmented out (pink). Approximately 12.5 μm of stroma from each group was focused on and viewed from above (insets), portraying the difference in fiber density. The stroma of WT cornea (1) contains a significantly higher concentration of elastic fibers compared to Fbn1^+/−^ stroma (2). All elastic fibers ran parallel to the surface of the cornea.

The image data obtained from the full thickness fiber quantification were used to create 3D reconstructions of WT and *Fbn1*^+/−^ corneas (Fig. 5). These models represent the first approximately 50 μm of stroma anterior to Descemet's membrane. The reconstructions also portray how the block was not cut perfectly parallel to the surface, resulting in stroma that runs obliquely from Descemet's membrane. An abundance of elastic fibers (colored gold) can be seen running throughout the stroma of both groups. Zooming into an approximately 12.5 μm region of stroma (Fig. 4, insets), and viewing from above, shows a clear difference in the concentration of elastic fibers, with WT cornea being more densely populated. Fibers from both groups run in all directions, primarily parallel to the surface of the cornea.

**Figure 6:**
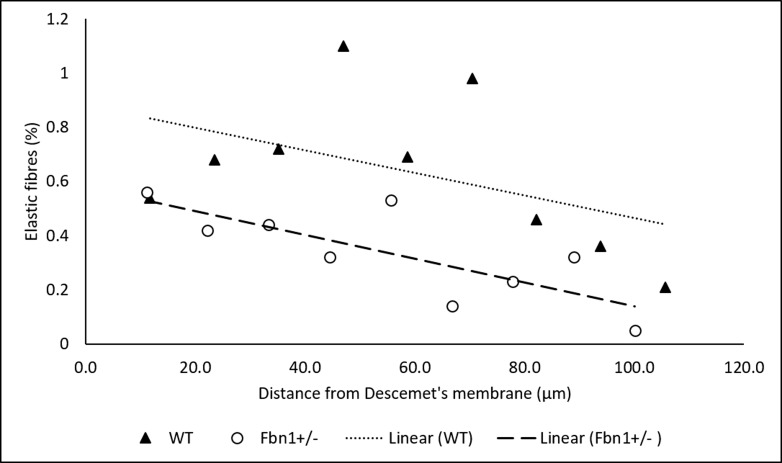
Volume of elastic fibers as a function of depth in the corneal stroma of one Fbn1^+/−^ and one WT mouse cornea. Each point on the graph represents approximately 250 serial en face images summed between that depth and the one before it in the graph. All the volume elements were rendered and the percentage of the volume element occupied by elastic fibers was calculated. The Fbn1^+/−^ cornea contained significantly less elastic fibers on average (approximately 50 %; P < 0.05) compared to the WT control, although this varied at different depths of stroma, with a 5-fold difference seen in the midstroma.

Approximately 2300 images were obtained from epithelium to Descemet's membrane in each corneal stroma, equating to a depth of 115 μm. The percentage of volume occupied by elastic fibers for each group of 250 images (equating to approximately 12.5 μm depth) was calculated for *Fbn1*^+/−^ and WT corneas (Fig. 6). Elastic fiber density was highest in the posterior *Fbn1*^+/−^ cornea before gradually decreasing to very low levels in the anterior stroma. The density of elastic fibers in the WT cornea showed a similar trend, but reached its highest point in the mid stroma before gradually decreasing toward the anterior. When comparing the two samples, the overall levels of elastic fibers were significantly lower (approximately 50%; *P* < 0.05) in the *Fbn1*^+/−^ cornea (*Fbn1*^+/−^ mean, 0.33% [± 0.16]; WT, mean 0.64% [± 0.27]), with the most dramatic difference being seen in the mid stroma, where the WT cornea had approximately 5 times more elastic fibers.

**Figure 7:**
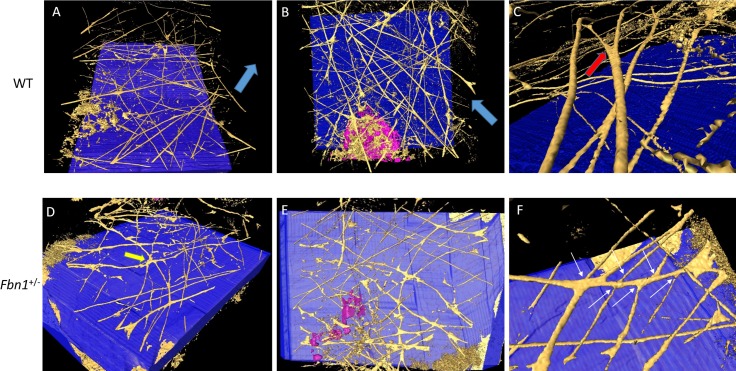
High resolution 3D reconstructions immediately anterior to Descemet's membrane (blue). WT (A–C) cornea shows an organized elastic fiber network (gold) running in a preferred direction (blue arrows), and occasionally bifurcating (red arrow). In contrast, fibers from Fbn1^+/−^ cornea (D–F) appear more disorganized, originating from a thickened area of tissue (yellow arrow) before travelling in several different directions. Fibers were seen to divide into many branches (white arrows).

Elastic fiber distribution was further analyzed at higher resolution in a region of stroma immediately anterior to Descemet's membrane (Fig. 7). Elastic fibers in the WT cornea were structurally organized, consisting of long fibers that travelled in straight lines with uniform thickness (Figs. 7A, 7B), occasionally bifurcating (Fig. 7C), with the two thinner fibers continuing to run in the same direction following the division. Elastic fibers in the *Fbn1*^+/−^ cornea appeared disorganized, running in multiple directions (Figs. 7D, 7E) when compared to the preferred orientation seen in WT cornea fibers. Five or six individual fibers were seen to originate from a broader region of elastic material at the center of the 3D reconstruction before travelling in several different directions (Fig. 7D). Furthermore, the elastic fiber in *Fbn1*^+/−^ cornea formed more extensive connections, with approximately 10 individual fibers seen sprouting from a thicker fiber (Fig. 7F).

### Transmission Electron Microscopy

**Figure 8:**
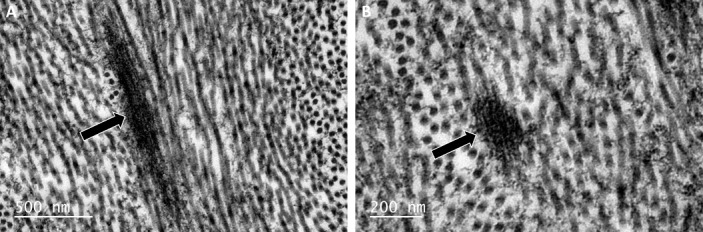
Transmission electron microscopy images of elastic fibers in the WT mouse cornea stained with tannic acid. Electron dense elastic fibers (black arrows) are orientated longitudinally (A) and transversely (B), with individual 10 to 12 nm microfibrils visible upon close inspection; these fibers can be easily distinguished from the surrounding collagen fibrils.

**Figure 9:**
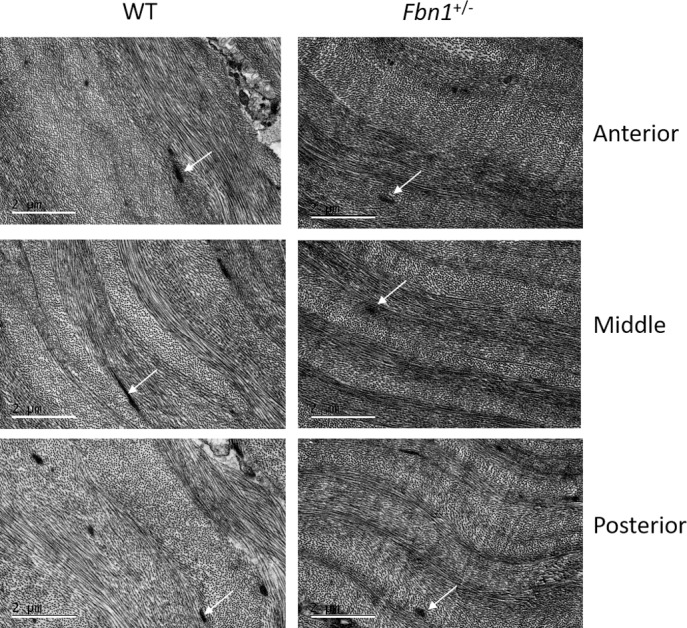
Transmission electron microscopy images obtained at various depths in the corneal stroma. Comparing anterior, middle, and posterior stroma of WT (left) and Fbn1^+/−^ (right) shows no obvious differences in ultrastructure. Both corneas appear well organized with comparable lamellae thickness. Elastic fibers are visible in each image (white arrows). Scale bars: 2 μm.

Elastic fibers in the mouse cornea of both groups were clearly visible in TEM micrographs (Fig. 8). The tannic acid-based staining method used stains elastic fibers as well as collagen.^26,27^ Elastic fibers were orientated longitudinally (Fig. 8A) and transversely (Fig. 8B), with individual 10 to 12 nm microfibrils visible within the transverse fibers; however, the 56 nm pseudoperiodicity seen in human corneal elastic fibers^8^ was not visible in the mouse elastic fibers. The diameter of the elastic fibers varied between 100 and 270 nm with an average diameter of approximately 187 nm in WT and approximately 145 nm in the *Fbn1*^+/−^ corneas (data not shown). Other than a higher quantity of elastic fibers in the WT cornea, no structural differences were apparent when comparing the anterior, middle, and posterior stroma of the two groups (Fig. 9). The stroma of the fibrillin-1–deficient mouse cornea contained organized lamellae, with comparable lamella thickness to WT cornea.

**Figure 10:**
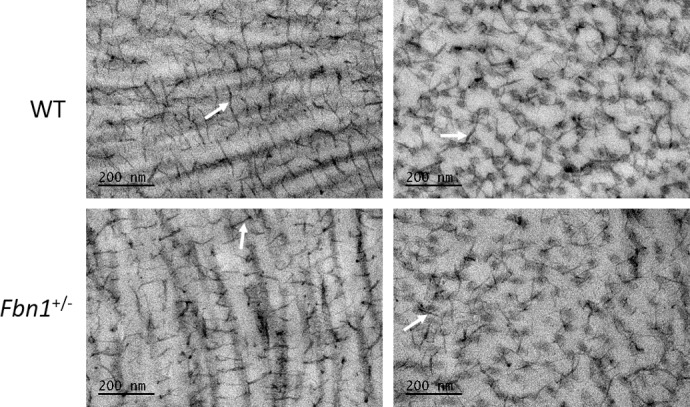
Proteoglycans stained with cupromeronic blue visualized with transmission electron microscopy. WT (top) and Fbn1^+/−^ (bottom) cornea, showing transversely (right) and longitudinally (left) oriented collagen fibrils with numerous associated GAG chains (white arrows) in the interfibrillar spaces, bridging the fibrils. No differences were apparent between the two groups.

**Figure 11:**
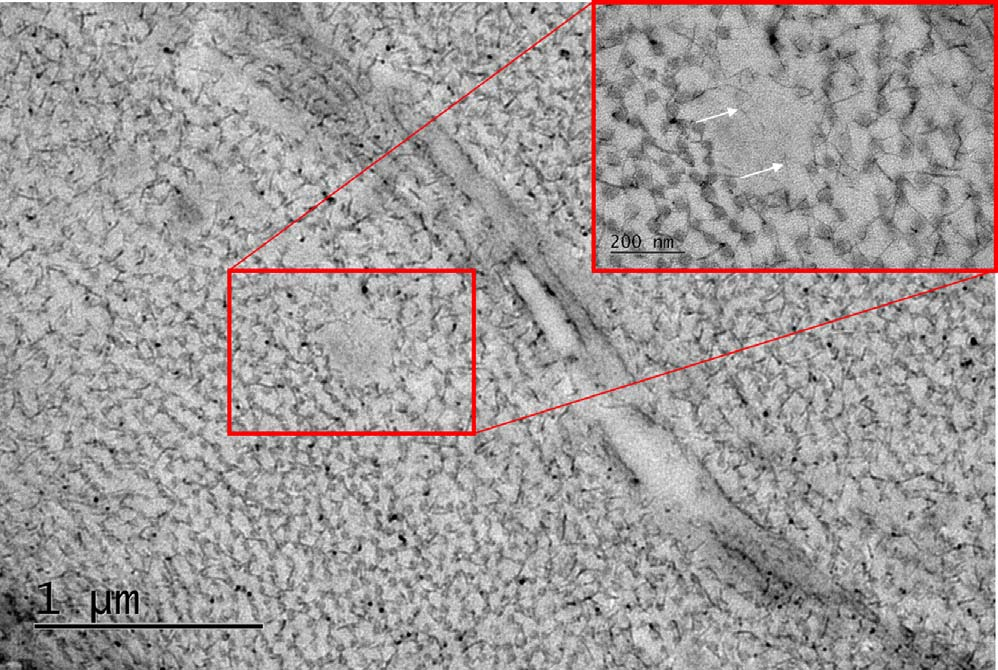
Proteoglycan-elastic fiber interaction shown with cupromeronic blue staining. An elastic fiber space in the stroma viewed at high magnification (inset) appeared to show proteoglycan filaments (white arrows) travelling into the transverse fiber and colocalizing with fibrillin-1 microfibrils.

Cupromeronic blue staining was done to compare proteoglycan quantity/distribution between the two groups (Fig. 10). Comparing the two sets of micrographs by eye suggested that there were no obvious differences in the quantity or spatial arrangement of proteoglycans between the two groups. Proteoglycan filaments were observed in the interfibrillar spaces, in association with collagen fibrils. These filaments formed bridges between transversely and longitudinally arranged collagen fibrils. Transverse elastic fibers were observed as circular blank spaces in the micrographs due to the omission of tannic acid from the staining protocol (Fig. 11). This assumption is based on the fact that the size and shape of the spaces closely match those of the elastic fibers seen in Figure 8. Observing the fiber spaces at high magnification indicated that there is a potential association between proteoglycan filaments and fibrillin-1, as suggested by long chondroitin sulfate/dermatan sulfate (CS/DS) glycosaminoglycan (GAG) chains appearing to travel into the elastic fiber (Fig. 11, inset).

### X-Ray Scattering

**Figure 12:**
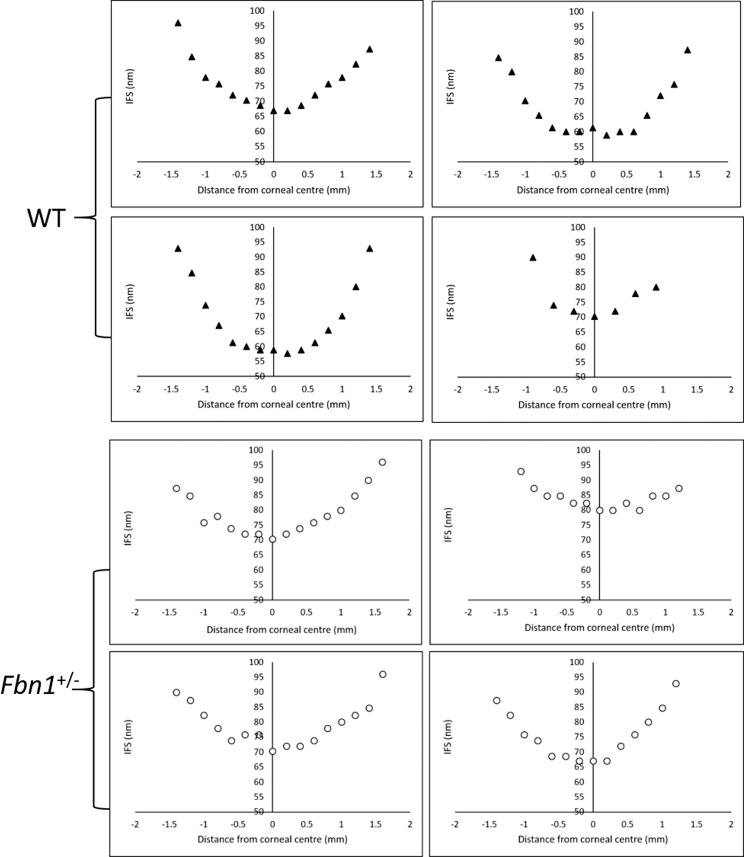
Radial plots of interfibrillar spacing (IFS) in WT and Fbn1^+/−^ mouse cornea. These graphs show the center–center average IFS in the center and periphery of WT and Fbn1^+/−^ corneas. Each plot represents a single cornea, and displays a “U” shaped distribution, with higher IFS at the periphery. At the center of the cornea, average IFS was significantly higher (P ≤ 0.05) in Fbn1^+/−^ corneas (71.9 nm) compared to WT equivalents (63.7 nm).

Average center–center fibril spacings for WT and *Fbn1*^+/−^ corneas are shown in Figure 12. In all specimens, IFS in the center of the cornea was clearly reduced compared to peripheral regions, where IFS increased by approximately 25% to 40%. This “U” shaped distribution also is seen when measuring mouse corneal fibril diameters.^25^ Corneal samples measured approximately 3 mm in diameter, indicating that each vertical scan was taken through the center, with the only exception being one WT cornea in which patterns were recorded at 0.3 mm intervals, as opposed to 0.2 mm intervals. Data from this sample suggested that the vertical transect was off center. D-periodicity at the center of each sample was consistent at 64 to 65 nm. Average IFS at the center of *Fbn1*^+/−^ corneas was higher (71.9 ± 5.6 nm) than the WT equivalents (63.7 ± 5.8 nm). Following the omission of the central IFS value from the off-center specimen, an independent *t*-test revealed that the difference in IFS was statistically significant (*P* ≤ 0.05), despite the low sample number (*n* = 3–4 for each group).

## Discussion

This study has provided evidence that the mgΔ^loxPneo^ mouse model for MFS is valuable for studying the corneal pathogenesis of the disease and the functional role of fibrillin-1 in the cornea. Importantly, quantification of elastic fibers in the fibrillin-1–deficient cornea showed a significant reduction (approximately 50%) in overall levels of fibrillin-1–containing elastic fibers throughout the entire thickness of the stroma compared to the WT control. These findings are consistent with studies from Lima et al.,^15^ who showed a diffused network of fibrillin-1–containing microfibrils in cultured foetal *Fbn1*^+/−^ fibroblasts when compared to WT. Similar findings were observed in human MFS patients, where decreased extracellular deposition of fibrillin-1 by fibroblasts has been reported.^28–30^

Optical coherence tomography microscopy image measurements revealed that central corneal thickness was significantly reduced in *Fbn1*^+/−^ mice compared to WT. Radius of curvature also was significantly different between the two groups, although there was no significant correlation between the two variables, which may be a symptom of underpowering the study. A significant reduction in central corneal thickness has been reported in human patients with MFS.^31–33^ A significant difference in keratometry readings also has been reported, suggesting a flattened cornea in MFS.^31,33–35^ Increased radius of curvature and increase in axial globe length in MFS cornea has been observed in previous studies.^34,36^ Therefore, based on these previous studies, and results obtained in the current study, we contend that the elastic fiber system has an important role in maintaining the curvature of the cornea. A reduction/malformation of taut elastic fibers in MFS would result in the cornea being pulled outwards at the periphery by an expanding globe following a release in internal stress, leading to a thin and flattened cornea. Elastin and elastin-free microfibril bundles are located in the sclera.^6,37^ This would imply an alteration in biomechanical properties in the sclera, as well as in the cornea, in MFS patients' eyes. With one of the main functions of the sclera being to maintain fixed axial dimensions to ensure a stable retinal image,^38^ and with absolute quantities of elastic tissue in the sclera unknown, it is likely that increased globe dimensions and subsequent flattened cornea associated with MFS are caused by a combination of biomechanical changes in the cornea and sclera. However, differences in corneal curvature in MFS patients, with no significant difference in axial length has been reported,^39^ which demonstrates the phenotypic variability of the disease. With no correlation between radius of curvature and central corneal thickness between *Fbn1*^+/−^ and WT mice detected, it is unlikely that corneal stretching alone is responsible for the observed changes in thickness. The biomechanical behavior of MFS cornea recently has been assessed in vivo using an ocular response analyzer, revealing a greater maximal deformation of the MFS cornea compared to control, which in turn indicates decreased resistance to bending.^39^ This evidence supports the proposal that elastic fibers have a pivotal role in providing the cornea with mechanical strength/elastic recoil, and maintaining corneal curvature.

By using SBF SEM to quantify elastic fibers, we are limited to analyzing an approximately 30 × 30 μm area of tissue throughout the stroma, with only 1 cornea from each group being analyzed due to the technical difficulty and time consuming nature of the technique. It is likely that the elastic fiber content varies slightly between each *Fbn1*^+/−^ cornea given the nature of the disease, ultimately resulting in a variable ratio of elastic fiber loss and fiber disorganization. The issue is complicated further by the fact that fibrillin-2 may compensate for the lack of fibrillin-1 proteins, as shown in mouse ciliary zonule.^40^ Nevertheless, we were able to show important alterations in the cornea of the mgΔ^loxPneo^ *Fbn1*^+/−^ mice, indicating that the mutant fibrillin-1 exerts a dominant-negative effect on the microfibrils. In comparison to human,^8^ elastic fibers are more densely populated in the mouse cornea, where they may be responsible for accommodating the increase in corneal curvature.

In the mouse cornea, elastic fibers are present throughout the entire stroma, whereas in human cornea they are highly concentrated in an approximately 10 μm region of stroma immediately anterior to Descemet's membrane, with concentration decreasing rapidly in the anterior direction.^8^ In accord with the studies of Hanlon et al.,^9^ our 3D volume rendering showed the presence of elastic fibers running in many directions parallel to the surface of the cornea and often bifurcating, similar to findings observed in the central human cornea.^8^ Comparisons of 3D reconstructions above Descemet's membrane indicated that elastic fibers in the *Fbn1*^+/−^ stroma were structurally different, appearing less ordered than the WT. Similarly, histologic examination of the lens capsule in human MFS patients revealed qualitative and quantitative differences, with significantly less fibrillin-1–containing microfibrils in addition to the presence of misshapen, irregular, and fragmented bundles.^41^

To our knowledge, this is the first study to demonstrate a reduction in elastic fiber density and microfibril disorganization in the cornea of a mouse model for MFS. In MFS, it is thought that the mutant protein that is produced has a dominant-negative effect over the normal protein, causing adverse activity on the deposition, stability, or function of the protein encoded by the normal allele of the *Fbn1* gene.^42^ Therefore, it is likely that the disorganized elastic fibers that are laid down in the mutant mouse cornea either contain the mutant protein, or their deposition has been diminished as the mutant protein has attempted to assemble with normal fibrillin-1 monomers, resulting in a structurally altered elastic system. A reduction in number and disorganization of elastic fibers would ultimately result in a biomechanically less responsive cornea, as the tissue relies more heavily on the collagen to deform reversibly under stress following the pull of extraocular muscles and changes in intraocular pressure.

The reduced number/disorganization of elastic fibers in *Fbn1*^+/−^ did not seem to have an adverse effect on overall structure of the cornea. Elastic fibers were similar in appearance to those previously reported in mouse cornea, with some fibers seen in close association with keratocytes (data not shown), suggesting a possible mechano-transductional role in the stroma.^9^ The slight reduction in mean fiber diameter in *Fbn1*^+/−^ cornea may be caused by less individual microfibrils making up the bundles. There currently are no reported structural changes associated with MFS.

Glycosaminoglycans side chains of the proteoglycan protein core provide hydration and swelling pressure to tissues, enabling them to withstand compression, in addition to mediating collagen diameter and IFS. Therefore, it was thought that differences in proteoglycan quantity/distribution may contribute to the differences in cornea thickness. No differences in proteoglycans were evident throughout the stroma of WT and *Fbn1*^+/−^ cornea. Electron dense filaments passing into an elastic fiber space suggest an association between CS/DS GAGs and fibrillin. The CS/DS proteoglycan decorin has been shown to be localized to fibrillin microfibrils in the skin.^43^ It has been suggested that CS/DS containing proteoglycans associate with fibrillin and contribute to microfibril organisation.^44^ Furthermore, fibrillin-1 and MAGP-1 (microfibril-associated glycoprotein-1) were shown to interact with decorin in cultured fetal bovine chondrocytes.^45^ In the rat eye, CS GAGs are associated with fibrillin-rich ciliary zonules.^46^ To our knowledge, this is the first study to indicate that GAGs colocalize with fibrillin in the cornea. It is likely that proteoglycans have an important role in the assembly and maintenance of elastic fibers.

X-ray scattering revealed that IFS was lowest at the central region of all specimens, increasing at the periphery, as observed previously in human^24,47^ and mouse^23^ corneas. Interfibrillar Bragg spacing was on average approximately 8 nm higher in the central region of *Fbn1*^+/−^ corneas, an unexpected result given that these corneas were significantly thinner than the WT's. Given that the corneas were fixed, the specific arrangement of collagen fibrils may have been altered.^48^ Center-to-center fibril spacing in the cornea is governed by several factors, mainly stromal hydration^49^ and proteoglycan composition/abundance.^50^ Thinner corneas accompanied by higher average IFS in the center indicates that *Fbn1*^+/−^ samples consist of fewer lamellae compared to WT's. This has similarities to keratoconic cornea, where x-ray scattering showed normal fibril spacings, indicating that the thin cornea in keratoconus is a result of reduced collagen quantity.^51^ Interestingly, earlier work by Patey et al.^52^ discovered a significant increase in collagen diameter and IFS in keratoconus as the disease progresses in severity, particularly in the middle and central stroma, leading to Pouliquen^53^ stating that this is the reason for stromal thinning and ectasia, since there are fewer lamellae. Therefore, findings from this current study showing increased IFS and thinner corneas in fibrillin-1–deficient animals are consistent with findings observed in keratoconus, suggesting that similar biochemical mechanisms may be active. As well as having a structural role, microfibrils forming elastic fibers are known to have a regulatory role in the extracellular matrix by binding cytokines (e.g., bone morphogenetic proteins and TGF-β).^54^ It may be possible that elastic fibers have a role in collagen biosynthesis, which could be negatively impacted by alterations in fibrillin-1 content.

It is probable that the elastic fibers carry out functions that are not apparent from ultrastructural observation, such as cell signaling and mechanotransduction in the extracellular matrix. Fibrillin is thought to have an important role in controlling TGF-β activation and signaling in the extracellular matrix, with studies showing dysregulation of TGF-β activity contributing to the pathogenesis of MFS, with excessive levels of active TGF-β in the matrix.^55–57^ Transforming growth factor–β is involved in many processes in the cornea, including keratocyte activation, myofibroblast transformation and proliferation, and wound healing.^58^

Overall, it is likely that elastic fibers have a multifunctional role in the cornea. This study, along with our previous study demonstrating the presence of elastic fibers in human cornea,^8^ should encourage more MFS–related research in the cornea. Further work is needed to elucidate the precise roles of elastic fibers in the cornea, as this is relevant for MFS pathogenesis, corneal biomechanics, development, and potentially other areas, such as extracellular matrix homeostasis.
